# KRAS(G12D) can be targeted by potent inhibitors via formation of salt bridge

**DOI:** 10.1038/s41421-021-00368-w

**Published:** 2022-01-25

**Authors:** Zhongwei Mao, Hongying Xiao, Panpan Shen, Yu Yang, Jing Xue, Yunyun Yang, Yanguo Shang, Lilan Zhang, Xin Li, Yuying Zhang, Yanan Du, Chun-Chi Chen, Rey-Ting Guo, Yonghui Zhang

**Affiliations:** 1 School of Pharmaceutical Science, Tsinghua-Peking Center for Life Sciences, Tsinghua University; Beijing Advanced Innovation Center for Human Brain Protection, Beijing, China; 2 Joint Graduate Program of Peking-Tsinghua-NIBS, School of Life Sciences, Tsinghua University, Beijing, China; 3 State Key Laboratory of Bio-catalysis and Enzyme Engineering, Hubei Collaborative Innovation Center for Green Transformation of Bio-Resources, School of Life Sciences, Hubei University, Wuhan, China; 4 Department of Biomedical Engineering, School of Medicine, Tsinghua-Peking Center for Life Science, Tsinghua University, Beijing, China

**Keywords:** Cancer, Cancer therapy

## Abstract

KRAS mutation occurs in nearly 30% of human cancers, yet the most prevalent and oncogenic KRAS(G12D) variant still lacks inhibitors. Herein, we designed a series of potent inhibitors that can form a salt bridge with KRAS’s Asp12 residue. Our ITC results show that these inhibitors have similar binding affinity with both GDP-bound and GTP-bound KRAS(G12D), and our crystallographic studies reveal the structural basis of inhibitor binding-induced switch-II pocket in KRAS(G12D), experimentally confirming the formation of a salt bridge between the piperazine moiety of the inhibitors and the Asp12 residue of the mutant protein. Among KRAS family proteins and mutants, both ITC and enzymatic assays demonstrate the selectivity of the inhibitors for KRAS(G12D); and the inhibitors disrupt the KRAS–CRAF interaction. We also observed the inhibition of cancer cell proliferation as well as MAPK signaling by a representative inhibitor (TH-Z835). However, since the inhibition was not fully dependent on KRAS mutation status, it is possible that our inhibitors may have off-target effects via targeting non-KRAS small GTPases. Experiments with mouse xenograft models of pancreatic cancer showed that TH-Z835 significantly reduced tumor volume and synergized with an anti-PD-1 antibody. Collectively, our study demonstrates proof-of-concept for a strategy based on salt-bridge and induced-fit pocket formation for KRAS(G12D) targeting, which warrants future medicinal chemistry efforts for optimal efficacy and minimized off-target effects.

## Introduction

The oncogenic impacts of the *KRAS* gene were first reported in 1980s, making *KRAS* one of the first identified oncogenes^1^. It is known that KRAS protein functions as a molecular switch: it responds to upstream EGFR activation and regulates the downstream MAPK and PI3K/mTOR pathways, eventually controlling cell proliferation, differentiation, and survival^2–5^.

Clinical data have implicated the driver mutations of the KRAS residue Gly12 (G12), and basic studies have shown that such mutations impair both this enzyme’s intrinsic and GTPase-activating protein (GAP)-stimulated GTP hydrolysis activity^6,7^, promoting oncogenesis. Despite nearly four decades of efforts, no direct KRAS inhibitor has been approved for medical use. There is consensus that the difficulty in developing direct KRAS inhibitors relates on the one hand to the picomolar affinity of GTP and GDP to KRAS (the intracellular concentrations of these metabolites are much higher), and on the other hand to an absence of suitable deep pockets for allosteric regulation.

One major breakthrough for KRAS inhibition was the discovery of an allosteric switch-II pocket (S-IIP) that is induced by covalent inhibitors of KRAS bearing the G12C driver mutation^8^. Studies have shown that induction of S-IIP results from covalent bond formation between the electrophilic acryloyl moieties of these inhibitors and the nucleophilic thiol moiety of the Cys residue at position 12^9–16^. These KRAS(G12C) inhibitors have shown promising results in recent clinical trials, although it is notable that they exclusively target KRAS in inactive state^9^. Despite these progresses with inhibitors of KRAS(G12C), the most prevalent and oncogenic G12 mutant variant is KRAS(G12D), which is estimated to impact more than 50% patients with pancreatic ductal adenocarcinoma^17^.

Various strategies have been testified for targeting KRAS(G12D), including those using indole-based small molecules to target a switch-I/II pocket, a compound (KAL-21404358) to target the P110 site, a pan-RAS inhibitor (compound 3144) to target the A59 site, and a cyclic peptide (KD2) to target KRAS(G12D)^18–21^. However, none of these molecules targets KRAS(G12D) with suitably low (micromolar) concentration. We speculated that a compound targeting the aspartic acid residue of KRAS(G12D) may somehow bind to KRAS(G12D) (similar to that formed between inhibitors and the cysteine residue of KRAS(G12C)), as a result may induce an allosteric pocket. This would perhaps enable pharmacological inhibition of this more prevalent oncodriver-mutation-bearing KRAS variant. We successfully developed a series of small molecule inhibitors of KRAS(G12D), which function by inducing an allosteric S-IIP and forming a salt bridge bonding with Asp12 residue, as confirmed by crystallographic studies. These inhibitors bind to both GDP-bound and GTP-bound KRAS(G12D), efficiently disrupt KRAS–CRAF interaction, but do not bind to wide type and G12C mutant KRAS. Our studies showed that they disrupted MAPK signaling, reduced tumor volume, and synergized with an anti-PD1 antibody in mouse xenograft models of pancreatic cancer. However, these inhibitors are also associated with off-target effects, likely due to binding and inhibiting some non-KRAS small GTPases, which warrants further structural optimizations.

## Results

### A salt bridge-based strategy for targeting KRAS(G12D) with a methyl-substituted piperazine inhibitor

Assuming that the α-carboxylic acid moiety of Asp12 is deprotonated under physiological conditions, we pursued a strategy based on the formation of a strong interaction (salt-bridge) between Asp12 and an alkyl amine moiety on an inhibitor. As the overall structures of the KRAS(G12C) and KRAS(G12D) variants are highly similar, we started our experiment based on a scaffold of the G12C inhibitor MRTX^22^ (Supplementary Fig. S1a). Removal of MRTX’s acryloyl moiety exposed a piperazine moiety that was predicted to position this alkyl amine in close enough proximity to Asp12 (2.2 Å) to support salt-bridge interaction (Supplementary Fig. S1b).

**Fig. 1:**
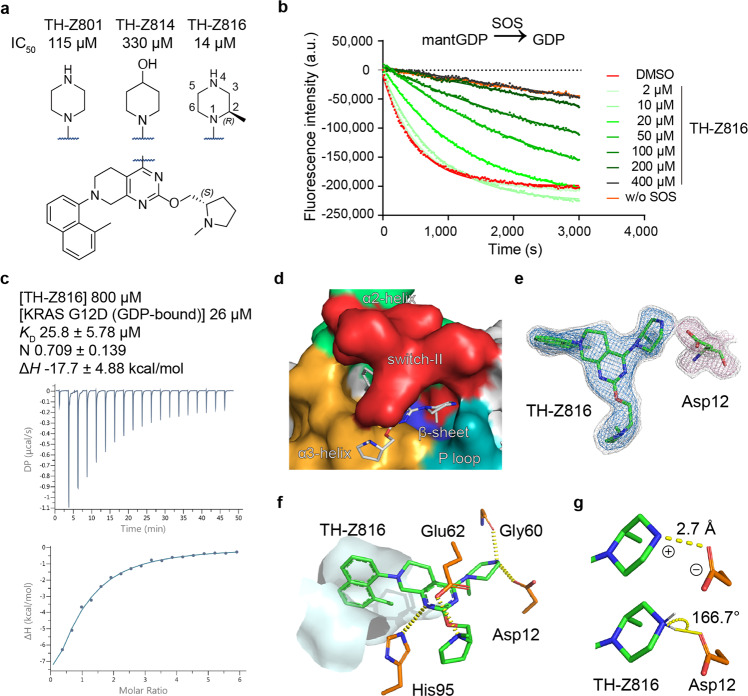
A piperazine-focused strategy for targeting KRAS(G12D) via salt-bridge interaction. **a** Chemical structures of TH-Z801 and TH-Z816 with exposed piperazines, structure of TH-Z814 with non-amine moiety. **b** Inhibitory activities of TH-Z816 on KRAS(G12D) measured by SOS-catalyzed nucleotide exchange assay with GDP as the incoming nucleotide. **c** ITC assay of the TH-Z816 (800 μM) and GDP-bound KRAS(G12D) (26 μM). **d** Crystal structure (PDB ID: 7EW9) of KRAS(G12D) bound to TH-Z816 (white stick). The binding pocket is formed by the α2-helix (green), switch-II (red), α3-helix (orange), P-loop (teal), and the central β-sheet (blue) of KRAS(G12D). **e** Fo-Fc maps of TH-Z816 (blue mesh, 2.5 σ; gray mesh, 1.5 σ) and Asp12 (pink mesh, 2.5 σ; gray mesh, 1.5 σ). **f** Interactions between TH-Z816 (green) and surrounding residues (orange). The hydrophobic sub-pocket is shown as a surface diagram. **g** The ionic bond (upper panel) and hydrogen bond (lower panel) between piperazine and Asp12.

Pursuing this, we synthesized TH-Z801 (Fig. 1a; Supplementary Fig. S2), which exerted relatively weak inhibition (IC_50_ = 115 μM), assessed based on the GDP/GDP exchange rate of KRAS(G12D) as catalyzed by SOS^9^. Additional structure-activity relationship (SAR) studies showed that replacement of the piperazine moiety with non-amine moieties dramatically decreased the inhibitory activity, supporting the functional contribution of a salt-bridge interaction in slowing down the GDP/GDP exchange rate (Fig. 1a; Supplementary Fig. S2).

Further chemical exploration focusing on piperazine substitution yielded TH-Z816 (wherein the piperazine was (R)-methyl-substituted), which had relatively strong inhibition activity (IC_50_ = 14 μM) (Fig. 1a, b). We further conducted isothermal titration calorimetry (ITC) assays to test whether TH-Z816 can bind directly to KRAS(G12D). Indeed, the detected binding affinity (K_D_) of TH-Z816 with KRAS(G12D) (GDP-bound) was 25.8 μM (Fig. 1c).

### A complex structure revealed an induced-fit pocket and confirmed the salt-bridge interaction

We solved a 2.13 Å co-crystal structure of KRAS(G12D) in complex with TH-Z816 (Supplementary Table S1). Our structure showed that TH-Z816 induced an allosteric pocket positioned under the KRAS(G12D) switch-II region (Fig. 1d); no such pocket was evident in the inhibitor-free structure (Supplementary Fig. S1c). This induced-fit pocket was located near the α2-helix, switch-II, α3-helix, the P-loop, and the central β-sheet of KRAS (Fig. 1d). Note that the pocket shape was quite similar (RMSD 0.293 Å, 148 to 148 atoms) to the MRTX-induced SII-P of KRAS(G12C) (Supplementary Fig. S1d).

Well-defined electron density clearly supported the conformation of TH-Z816 and KRAS(G12D) (Fig. 1e). Specifically, the naphthyl moiety of TH-Z816 embedded deeply into the pocket and formed hydrophobic interactions with residues Val9, Met72, Phe78, Gln99, Ile100, and Val103 (Supplementary Fig. S1e). There are also four pairs of polar interactions between TH-Z816 and residues His95, Glu62, Gly60, and Asp12 (Fig. 1f). Note that an ionic bond and a hydrogen bond together comprise the anticipated salt-bridge: on one hand, the positively charged piperazine moiety of TH-Z816 and negatively charged side chain of Asp12 are close enough to support an ionic bond (Fig. 1g); on the other hand, the measured bond angle (166.7°) and length (2.7 Å) supports the presence of a hydrogen bond between these two moieties (Fig. 1g).

### A cyclization strategy to improve inhibitory potency by optimizing Δ*S*

**Fig. 2:**
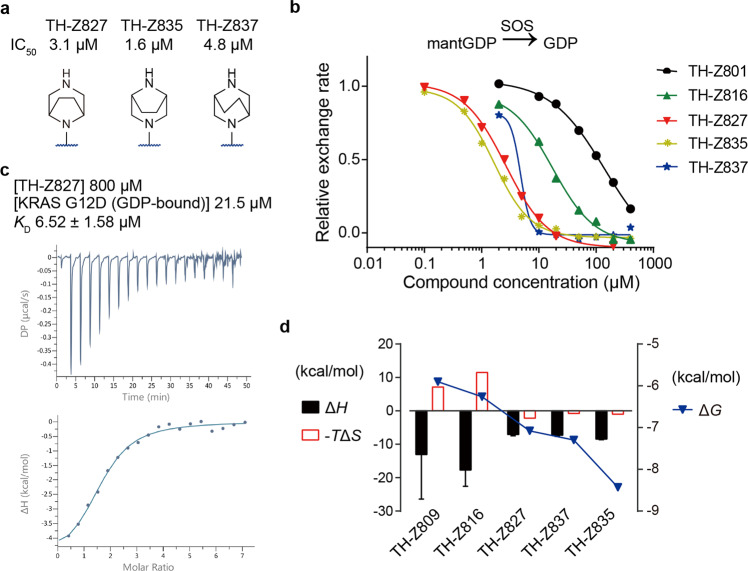
A cyclization strategy to improve inhibitory activities and binding potency. **a** Chemical structures of bicyclic compounds TH-Z827, TH-Z835, and TH-Z837. **b** Inhibitory activities of these bicyclic compounds on KRAS(G12D) measured by SOS-catalyzed nucleotide exchange assay with GDP as the incoming nucleotide. **c** ITC assay of the TH-Z827 (800 μM) and GDP-bound KRAS(G12D) (21.5 μM). **d** Δ*G*, Δ*H,* and Δ*S* analysis of compounds tested by ITC assays. For each compound, Δ*H* and -*T*Δ*S* values are presented referring to the left axis, while the ΔG value is presented referring to the right axis.

Further optimization of TH-Z816 was guided by the following thermodynamic rule, Δ*G* = Δ*H* – *T*Δ*S*^23,24^. We sought to optimize Δ*S* by restraining the conformational freedom of the piperazine moiety. Given the axial position of the methyl group, we employed a cyclization strategy based on TH-Z816 and generated the bicyclic compound TH-Z827 (Supplementary Fig. S3a). As TH-Z827 showed a more than eight-fold increase in potency (IC_50_ = 3.1 μM), we subsequently adopted a Δ*S*-focused cyclization strategy and designed additional bicyclic compounds (Fig. 2a), among which the most potent one was TH-Z835 (IC_50_ = 1.6 μM) (Fig. 2b).

We next conducted ITC assays to measure the binding affinity of these bicyclic compounds to KRAS(G12D) in the presence of GDP (Fig. 2c; Supplementary Fig. S3b, c). Compared with TH-Z816, bicyclic compounds TH-Z827, TH-Z835, and TH-Z837 had unfavorable Δ*H* changes yet favorable Δ*S* changes (Fig. 2d), indicating that the binding affinity (Δ*G*) increase of these bicyclic compounds can be attributed to Δ*S* improvement.

### G12D inhibitors bind to both GDP-bound and GMPPNP-bound KRAS with similar affinities

Previous studies have shown that KRAS exists in cells in both GTP-bound and GDP-bound states^13,25–30^. A molecular docking analysis of our KRAS(G12D) inhibitors indicated that the absence of an acryloyl moiety in the inhibitors results in sufficient space (4.7 Å) for the γ-phosphate of GTP, while the salt-bridge interaction between Asp12 of KRAS and the piperazine moiety of our inhibitors is maintained (Supplementary Fig. S4a).

**Fig. 3:**
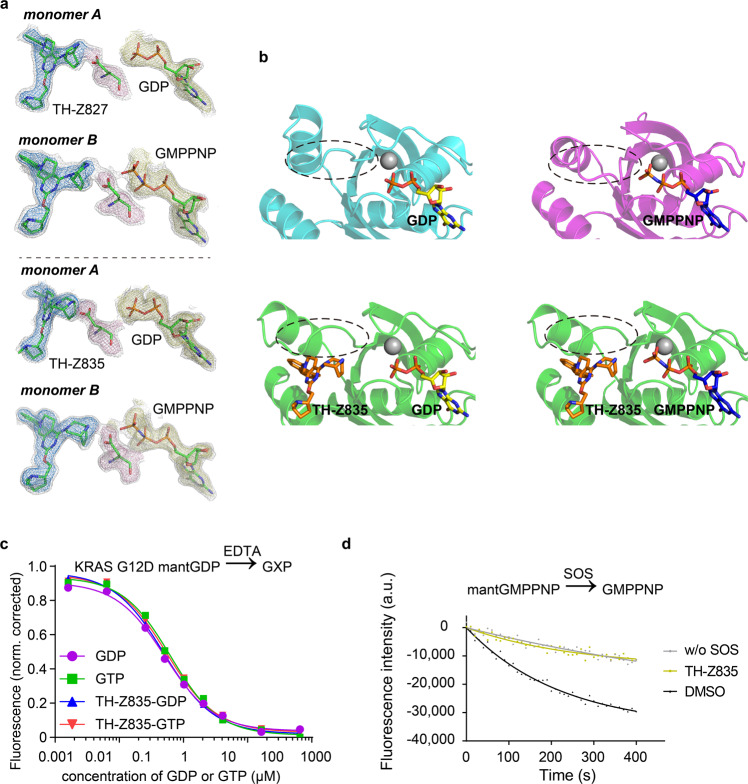
G12D inhibitors bind to both GDP-bound and GMPPNP-bound KRAS. **a** Upper panel: Fo-Fc map of TH-Z827, Asp12, and GDP (PDB ID: 7EWA, monomer A). Fo-Fc map of TH-Z827, Asp12, and the GTP analog GMPPNP (PDB ID: 7EWA, monomer B). Lower panel: Fo-Fc map of TH-Z835, Asp12, and GDP (PDB ID: 7EWB, monomer A). Fo-Fc map of TH-Z835, Asp12, and GMPPNP (PDB ID: 7EWB, monomer B). In all graphs, the 1.5 σ Fo-Fc maps of all shown elements are shown in gray mesh. The color scheme of 2.5 σ Fo-Fc maps is: blue for inhibitors, pink for Asp12, and yellow for nucleotides. **b** KRAS(G12D) conformational change analysis for drug-free GDP-bound protein (PDB: 4EPR), drug-free GMPPNP-bound protein (PDB: 5USJ), TH-Z835 and GDP-bound protein (PDB: 7EWB, chain A), and TH-Z835 and GMPPNP-bound protein (PDB: 7EWB, chain B). **c** EDTA-mediated competition between fluorescently labeled mantGDP loaded on KRAS and free nucleotide (GDP or GTP). The experiment was carried out with KRAS(G12D) alone or with KRAS(G12D) treated with TH-Z835. **d** Inhibitory activity of TH-Z835 measured by SOS-catalyzed nucleotide exchange assays with GMPPNP as the incoming nucleotide.

To reveal whether these inhibitors bind to the GTP-bound KRAS(G12D), we first treated the purified protein with GMPPNP (a stable analog of GTP) and EDTA, following a well-established nucleotide-exchange protocol^8,9^. We next aimed at crystallizing GMPPNP-containing KRAS(G12D) in complex with these more active inhibitors TH-Z827 and TH-Z835, and successfully solved a 2.25 Å co-crystal structure of KRAS(G12D) in complex with TH-Z827 (PDB ID: 7EWA; Fig. 3a; Supplementary Table S1). This crystal structure of KRAS(G12D) was trimeric, with monomer A bound to GDP and TH-Z827, monomer B bound to GMPPNP and TH-Z827, and monomer C bound to GMPPNP. We also solved a co-crystal structure of KRAS(G12D) in complex with TH-Z835 (PDB ID: 7EWB; Fig. 3a; Supplementary Table S1). In these two inhibitors–KRAS(G12D) structures, the electron densities were well-defined for inhibitors, Asp12, and both GMPPNP and GDP. Our data confirmed that both TH-Z827 and TH-Z835 are able to interact with Asp12 and form a salt bridge for both GDP-bound and GMPPNP-bound KRAS(G12D) (Fig. 3a). It is noted that in the inhibitor-free GMPPNP-bound state, switch II more tightly bound to the γ-phosphate. However, the binding of TH-Z835 shifted the conformation of switch II towards the inhibitor-free GDP-bound conformation (analysis shown in Fig. 3b).

We next conducted ITC assays to measure the binding affinities between G12D inhibitors and GMPPNP-bound KRAS (G12D) (Supplementary Fig. S4b). The detected binding affinities for each of the tested compounds were within a narrow range for both the GDP-bound and the GMPPNP-bound forms (Supplementary Fig. S4c), and EDTA-catalyzed nucleotide exchange assays that test the GDP/GTP binding preference of KRAS(G12D) supported our ITC results, showing no GDP/GTP binding preference for KRAS(G12D) in the presence of our inhibitors (Fig. 3c). In contrast, KRAS(G12C) had a significantly lower affinity for GTP than for GDP in the presence of the G12C inhibitor MRTX (Supplementary Fig. S4d), which was consistent with the previous reports^9^. We also conducted SOS-catalyzed nucleotide exchange assays of KRAS(G12D), which experimentally confirmed that our inhibitor TH-Z835 does inhibit both mantGMPPNP/GPPNP exchange and GPPNP/mantGMPPNP exchange (Fig. 3d; Supplementary Fig. S4e).

### Mutant selectivity of the KRAS(G12D) inhibitor TH-Z827

**Fig. 4:**
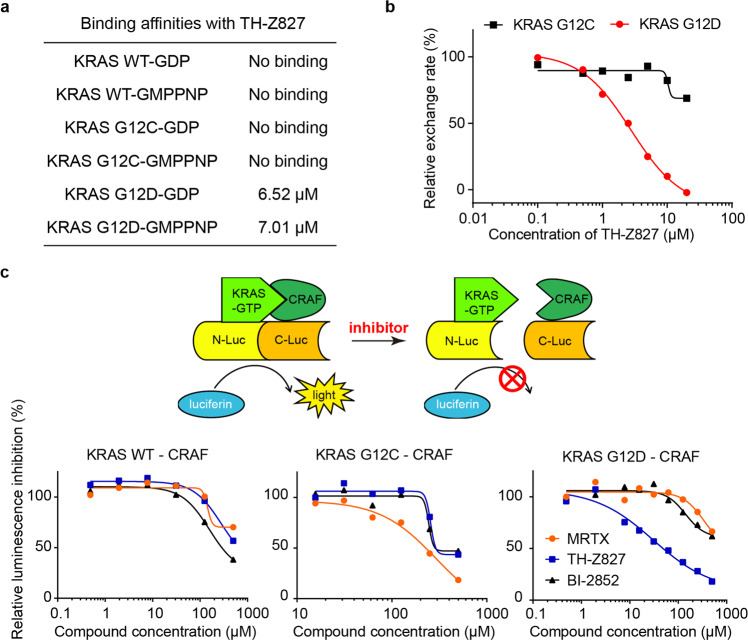
Mutant-selectivity of the KRAS(G12D) inhibitor TH-Z827. **a** Binding affinities between TH-Z827 and GDP- or GMPPNP-bound KRAS (WT, G12C, or G12D). **b** SOS-catalyzed KRAS(G12C) or KRAS(G12D) nucleotide exchange assays in the presence of TH-Z827. **c** Principle of the split-luciferase reporter assay that detects the KRAS–CRAF interaction in lysates from HEK 293T cells, with or without TH-Z827. Two other compounds (the G12C inhibitor MRTX and the pan-RAS inhibitor BI-2852) were included as controls.

We next studied the mutant selectivity of our G12D inhibitors using ITC assays and did not detect measurable binding for TH-Z827 with KRAS(WT) or with KRAS(G12C), no matter whether the proteins were GDP-bound or GMPPNP-bound **(**Fig. 4a; Supplementary Fig. S5). SOS-catalyzed nucleotide exchange assays also showed that TH-Z827 exerted a nearly 10-fold stronger inhibition for KRAS(G12D) than for KRAS(G12C) (IC_50_ = 2.4 μM vs IC_50_ = 20 μM) (Fig. 4b).

After confirming the mutant-selectivity of targeting KRAS, we further tested whether our inhibitor could selectively block the interactions of various KRAS mutants with its effector protein. CRAF is a well-studied KRAS effector protein, and the KRAS–CRAF interaction is known to promote MAPK signal transduction^31–33^. We established a split-luciferase reporter system using HEK 293T cells (Fig. 4c). Upon doxycycline treatment, the cells express both full-length KRAS (fused to N-luciferase) and a truncated CRAF variant (comprising residue 50–220) fused to C-luciferase. After lysis, the supernatant was incubated with our inhibitor and the substrate luciferin. When KRAS binds to CRAF, luciferase complementation results in the emission of a luminescence signal. The results showed that TH-Z827 blocked the KRAS(G12D)–CRAF interaction with an IC_50_ value of 42 μM but did not affect CRAF’s interaction with KRAS(WT) or KRAS(G12C) at this concentration (Fig. 4c).

### Inhibition of KRAS(G12D) mutant cell lines

**Fig. 5:**
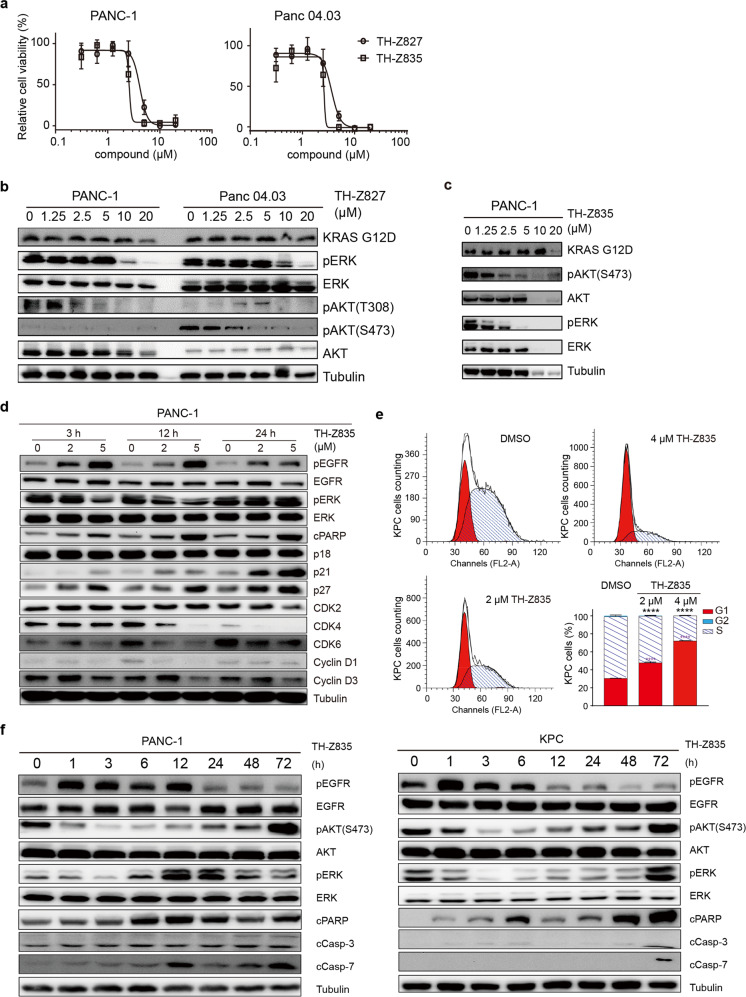
Inhibition of KRAS(G12D) mutant cell lines by TH-Z827 and TH-Z835. **a** Viability assays of PANC-1 and Panc 04.03 cells treated with indicated concentration of TH-Z827 or TH-Z835 for 24 h. **b** Immunoblotting analysis of ERK and AKT phosphorylation status in PANC-1 (left panel) and Panc 04.03 cells (right panel) treated with TH-Z827 for 3 h. **c** Immunoblotting analysis of ERK and AKT phosphorylation status in PANC-1 cells treated with TH-Z835 for 3 h. **d** Immunoblotting against RTK signaling and cell cycle marker proteins in PANC1 cells after 3-, 12- or 24 h treatment with the indicated concentrations of TH-Z835. **e** Percentages of KPC cells in the G1, S, and G2 phases after treated with TH-Z835 for 24 h (G2 phase on the top, less than 2%). Data are shown as means ± SEM. *n* = 3, two-tailed Student’s *t*-test, **** *P* < 0.0001. **f** Immunoblotting of EGFR, ERK, AKT phosphorylation, cleaved PARP (cPARP), cleaved Caspase-3 (cCasp-3), and cleaved Caspase-7 (cCasp-7) in PANC-1(left panel) and KPC (right panel) cells treated with TH-Z835 (5 μM).

The promising results for the inhibitory activity and mutant-selectivity from the ITC and KRAS–CRAF interaction assays motivated us to evaluate the potential anti-cancer effects of TH-Z827. The KRAS G12D mutation is the most prevalent mutation form that drives the most prevalent type of pancreatic cancer^34^. In two pancreatic cancer cell lines bearing the KRAS G12D mutation (PANC-1 and Panc 04.03), TH-Z827 conferred anti-proliferative effects with IC_50_ values of 4.4 and 4.7 μM, respectively (Fig. 5a). Treatment with TH-Z827 also reduced the levels of pERK and pAKT in PANC-1 and Panc 04.03 cells (Fig. 5b), confirming that TH-Z827 prevented the activation of MAPK and PI3K/mTOR signaling. We observed that TH-Z835 reduced the pERK level in PANC-1 cells with an IC_50_ value less than 2.5 μM (Fig. 5c), which was more potent than TH-Z827 (Fig. 5b). We next performed 2D adherent, 3D non-adherent, and colony formation assays and observed anti-proliferative effects of TH-Z835 for two KRAS(G12D)-bearing pancreatic cancer cell lines: PANC-1 and KPC (KrasLSL.G12D/+; p53R172H/+; PdxCretg/+). It was notable that the IC_50_ values of TH-Z835 in the colony formation assay were less than 0.5 μM (Supplementary Fig. S6a, b).

Further examination of the inhibitor-treated PANC-1 cells revealed increased protein levels of p21 and p27, as well as decreased levels of CDK2/4/6 and Cyclin D1, indicating that TH-Z835 induces arrest at the G1 phase of the cell cycle (Fig. 5d). Consistent with these western blotting results, flow cytometry also showed an increased population of PANC-1 cells in G1 phase and a decreased population in S phase as compared with the DMSO control group (Fig. 5e). We also used flow cytometry to evaluate apoptosis induction in TH-Z835-treated PANC-1 and KPC cells and found significantly increased proportions of Annexin V-positive cells (Supplementary Fig. S6c, d). We confirmed this finding by immunoblotting which showed that treatment with TH-Z835 led to increased levels of the apoptosis markers including cleaved PARP, cleaved caspase-3, and cleaved caspase-7 (Fig. 5f).

### TH-Z835 is associated with off-target effect, likely due to targeting non-KRAS small GTPases

**Fig. 6:**
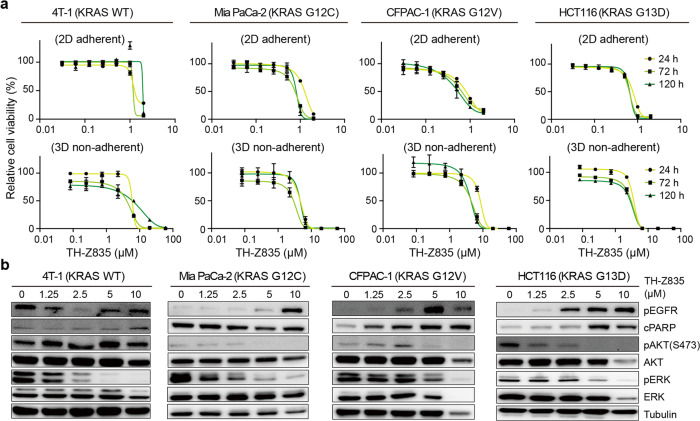
Anti-proliferative effects and signaling inhibition of TH-Z835 on non-KRAS(G12D) cells. **a** Cell viability assays of 4T1, MIA PaCa-2, CFPAC-1, and HCT116 cells with indicated concentration of TH-Z835 for 24 h, 72 h, or 120 h in 2D adherent assays (upper panel) and 3D non-adherent assays plates (lower panel). **b** Immunoblotting of EGFR, ERK, AKT phosphorylation, and cPARP in 4T1, MIA PaCa-2, CFPAC-1, and HCT116 cells treated with TH-Z835 for 3 h.

We next tested the antiproliferative effects of TH-Z835 in other non-G12D mutant cancer cell lines, including 4T1 (KRAS(WT)), MIA PaCa-2 (KRAS(G12C)), CFPAC-1 (KRAS(G12V)), and HCT116 (KRAS(G13D)) cells. Different from our expectations based on data of our in vitro protein assay for the KRAS family proteins, these assays showed that TH-Z835 also conferred anti-proliferative effects (Fig. 6a), reduced the pERK and pAKT levels in these cells (Fig. 6b) and induced apoptosis (Supplementary Fig. S7a–d), suggesting off-target effects. One likely explanation is that TH-Z835 targets some non-KRAS small GTPases, especially given that the switch-II regions of these proteins share structural similarity to those of KRAS proteins. It should also be noted that cell proliferation and MAPK signaling induction in diverse cancers have been shown to be regulated by other Ras superfamily proteins, including members of the Rho, Ran, Arf, and Rab families^35^.

### In vivo antitumor activity and combination with PD-1 therapy

**Fig. 7:**
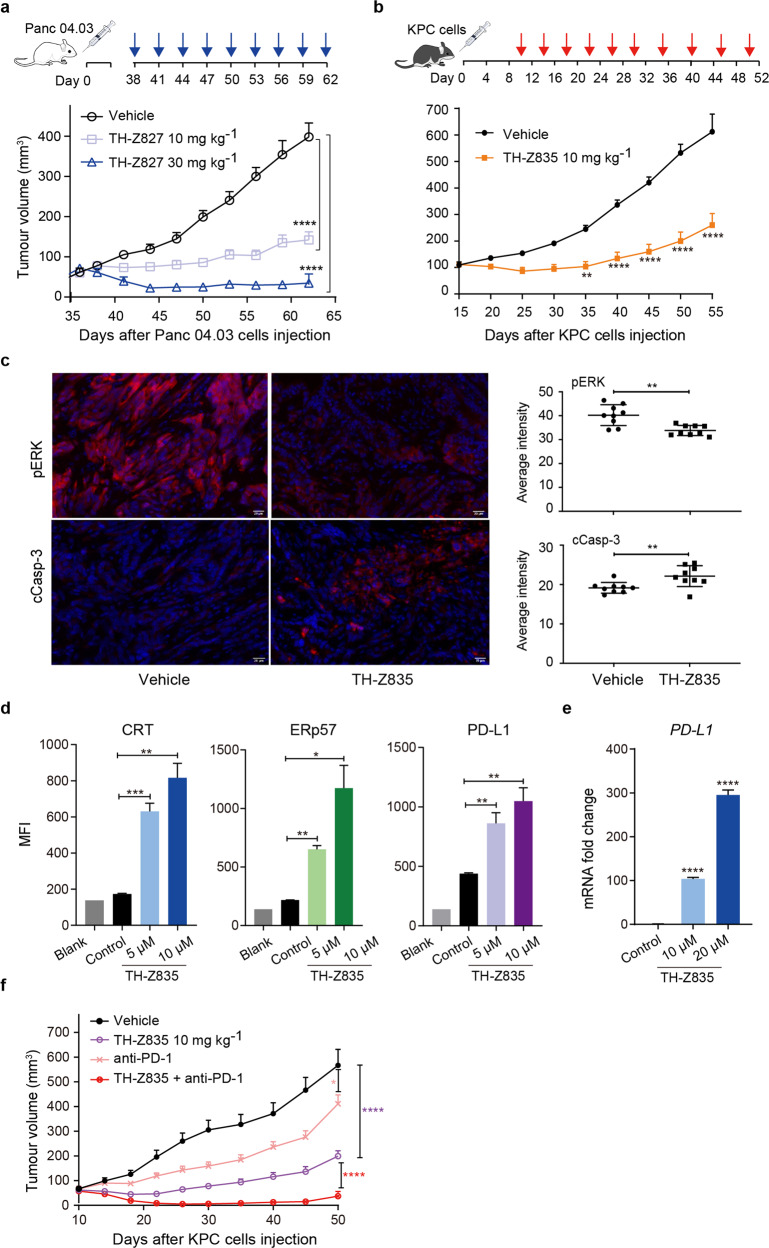
Anti-tumor effects of the KRAS(G12D) inhibitors alone and in combination with anti-PD-L1 antibody. **a** Nude mice injected with Panc 04.03 cells at Day 0 were later intraperitoneally injected with TH-Z827 (at 10 mg/kg or 30 mg/kg) according to the indicated dosage schedule (blue arrow). The tumor volumes (means ± SEM, *n* = 10) were measured with digital calipers and assessed using one-way ANOVA followed by Dunnett’s test, **** *P*_adj_ < 0.0001. **b** Mice were injected with KPC cells at Day 0 and TH-Z835 at Day 10. The tumor volumes (means ± SEM, *n* = 10) were assessed using one-way ANOVA followed by Dunnett’s test, ** *P*_adj_ < 0.01, **** *P*_adj_ < 0.0001. **c** Left panel: Immunofluorescence (IF) analysis of pERK and cleaved Caspase-3 in tumor sections from C57BL/6 mice (**b**) treated with TH-Z835 or vehicle. Scale bar, 20 μm. Right panel: quantification of IF-positive staining. Data are shown as means ± SEM. *n* = 9, two-tailed Student’s *t*-test, ** *P* < 0.01. **d** Flow cytometry analysis of the immunogenic cell death (ICD) markers CRT and ERp57 and the immune checkpoint protein PD-L1 on the surface of KPC cells after 24 h treatment with TH-Z835. Data are shown as means ± SEM (*n* = 3), two-tailed Student’s *t*-test, * *P* < 0.05, ** *P* < 0.01, *** *P* < 0.001. **e** mRNA expression level of *PD-L1* in PANC-1 cells after 24 h treatment with TH-Z835. Data are shown as means ± SEM (*n* = 3), two-tailed Student’s *t*-test, **** *P* < 0.0001. **f** C57BL/6 mice were injected with KPC cells at Day 0, after which TH-Z835, anti-PD-1 antibody, or a combination therapy (10 mg/kg TH-Z835 and 100 μg per dose anti-PD-1 antibody) was intraperitoneally administered. The tumor volumes (means ± SEM, *n* = 5) were assessed using one-way ANOVA followed by Dunnett’s test, * *P*_adj_ < 0.05, **** *P*_adj_ < 0.0001.

Our in vivo testing of KRAS(G12D) inhibitors was conducted in two xenograft pancreatic tumor models: BALB/c nude mice subcutaneously inoculated with Panc 04.03 cells and C57BL/6 mice inoculated with KPC cells. In the nude mice model, TH-Z827 significantly reduced the tumor volumes compared to the vehicle control, and did so in a dose-dependent manner (Fig. 7a). However, an intraperitoneal dosing of 30 mg/kg TH-Z827 resulted in observed weight loss, again suggesting the potential of off-target effects (Supplementary Fig. S8a). We next tested the antitumor activity of TH-Z835 in the C57BL/6 mice model. The tumor volume was also reduced compared with the vehicle group (Fig. 7b). In addition, immunohistochemical analysis of tumor sections revealed an increased expression of cleaved caspase-3 and a decreased expression of pERK1/2 (Fig. 7c; Supplementary Fig. S8b), indicating that TH-Z835 induces apoptosis and inhibits MAPK signaling in vivo.

Recent studies showed that besides its function in cancer cells, KRAS oncogenic signaling can orchestrate the immune status of the tumor microenvironment^36–38^. Specifically, studies using murine pancreatic cancer models showed that KRAS can drive immune evasion (characterized by scant intratumoural CD8^+^ T cells)^39^. In addition, activated MAPK signaling may also be involved in the immunosuppressive tumor microenvironment^40^. We first tested the effect of TH-Z835 treatment on KPC cells and found increased *PD-L1* mRNA expression and increased levels of the immunogenic cell death markers CRT and ERp57 on the cell surface (Fig. 7d, e; Supplementary Fig. S8c). We next tested the efficacy of a combination therapy comprising TH-Z835 and an anti-PD-1 antibody for C57BL/6 mice inoculated with KPC cells. Indeed, a combination treatment led to a statistically significant decrease in tumor volume as compared to either of the mono-therapies (Fig. 7f). This synergism could also be observed in a combination therapy comprising TH-Z827 and the anti-PD-1 antibody (Fig. S8d).

## Discussion

Aiming to develop inhibitors for KRAS(G12D), a more common KRAS mutant variant, we synthesized a series of small molecules that can form a salt bridge with Asp12 residue, mimicking the acryloyl–cysteine interaction of KRAS(G12C) and its inhibitors, and characterized their activities both in vitro and in vivo. Unlike covalent KRAS(G12C) inhibitors that selectively bind to GDP-bound proteins, both our computational and ITC investigations suggest that these inhibitors that can form salt bridge bind to both GDP-bound and GTP-bound KRAS(G12D). We also solved crystal structures of KRAS(G12D) with series of potent inhibitors (TH-Z816, TH-Z827, and TH-Z835). Our structural data confirmed that these inhibitors can induce the formation of an allosteric pocket under the KRAS(G12D) switch-II region, similar to reported findings for the covalent KRAS(G12C) inhibitors^8,9,13,15^. In addition, the crystal structures revealed that these inhibitors are able to bind to the GMPPNP-bound KRAS(G12D). That is, whereas steric clashing renders the G12C inhibitors incapable of targeting GTP-bound KRAS(G12C)^9^, our salt-bridge forming inhibitors have sufficient space to target Asp12 for both GDP-bound and GTP-bound KRAS(G12D). Notably, the discovery of G12C inhibitors might result in a stereotype that targeting the GDP-bound (inactive) KRAS may be a more viable approach than targeting the GTP-bound (active) KRAS^5^. Now, using the inhibitors developed in the present study, it should be possible to launch hypothesis-driven basic and translational investigations about the differential impacts and therapeutic consequences of targeting active KRAS mutant.

We detected no binding or inhibitory effects of our compounds towards the KRAS(WT) or KRAS(G12C) proteins, whereas our data show that these KRAS(G12D) inhibitors can efficiently disrupt the KRAS–CRAF interaction. These molecules also disrupt the activation of MAPK and PI3K/mTOR signaling in diverse cancer cells and display anti-proliferative and anti-tumor effects. However, despite this apparently clear picture from our in vitro work, we stress that this efficacy is not fully dependent on KRAS mutation status: our data from assays with diverse cancer cells bearing WT KRAS revealed a more complex interaction pattern. Our experiments with xenograft model of pancreatic tumors showcase the very promising efficacy and synergistic potential for combination therapy of TH-Z835; nevertheless, our observation of weight loss again suggested off-target impacts. A very likely explanation is that the inhibitors bind to and inhibit Rho, Ran, Arf, and/or Rab subfamily proteins that also function in regulating cancer cell proliferation and MAPK signaling. In sum, our study has demonstrated proof-of-concept for strategy based on the formation of a salt-bridge and induced-fit pocket to achieve KRAS(G12D) inhibition, which warrants future medicinal chemistry efforts to obtain better specificity and optimal efficacy.

## Methods

### Protein expression and purification

The gene encoding KRAS(G12D) (residues 1–169) was chemically synthesized and cloned into pET28a vector with *Nde*I and *Xho*I restriction sites. A construct for the recombinant KRAS(G12D) was transformed into *E. coli* BL21 (DE3). After the bacterial growth to an OD_600_ of 0.6, induction was carried out using 1 mM isopropyl-β-D-thiogalactopyranoside (IPTG) (16 °C for 24 h). The cells were lysed and recombinant KRAS(G12D) was purified using a HisTrap HP (GE Healthcare, 29-0510-21) column. The hexahistidine tag was then removed by TEV-protease and crude protein was further purified. The mature protein was concentrated to 3.5 mg/mL, and the molecular mass of protein was determined by SDS-PAGE (~21 kDa). The KRAS protein sequence is ‘MTEYKLVVVG ADGVGKSALT IQLIQNHFVD EYDPTIEDSY RKQVVIDGET CLLDILDTAG QEEYSAMRDQ YMRTGEGFLC VFAINNTKSF EDIHHYREQI KRVKDSEDVP MVLVGNKCDL PSRTVDTKQA QDLARSYGIP FIETSAKTRQ GVDDAFYTLV REIRKHKEK’. SOS, KRAS(WT), and KRAS(G12C) were expressed and purified in a similar way as KRAS(G12D). The plasmid of SOS was provided by Professor Niu Huang of National Institute of Building Sciences.

### X-ray crystallography

The protein was further concentrated to 40 mg/mL for the X-ray crystallography study. For TH-Z816, KRAS(G12D) protein was directly used for the following procedure. For TH-Z827 and TH-Z835, the endogenous GDP of KRAS(G12D) was exchanged with GMPPNP catalyzed by EDTA. Next, using the vapor-diffusion method, thin plates were observed after a week at 20 °C under the crystallization conditions of 0.2 M sodium acetate, 0.1 M Tris, pH 8.5, 26% (w/v) PEG 3350. To obtain the complex structures, the protein crystals were soaked into 2 mM inhibitor for 6 h. The soaked crystals were cryoprotected in the mother liquor supplemented with 10% glycerol prior to flash-freezing. The X-ray diffraction data were obtained at the in-house beamline BRUKER D8 VENTURE at Hubei University. Datasets were initially processed with PROTEUM3 v2020.6, solved by molecular replacement using Phaser with KRAS (PDB ID: 4EPR), and refined to the indicated statistics using PHENIX 1.10.1-2155 and Coot 0.8.3^41^. The figures were drawn using PyMOL.

### EDTA-catalyzed nucleotide exchange

Endogenous nucleotides in KRAS were exchanged with GDP (Sigma, G7127), GMPPNP (Sigma, G0635), mantGDP (Jena Bioscience, NU-204S), or mantGMPPNP (Jena Bioscience. NU-207S) using a previously reported EDTA-catalyzed procedure^42,43^. Briefly, KRAS(G12D) protein (10 μM) was incubated with incoming nucleotide (200 μM) and EDTA (2.5 M) for 1.5 h at room temperature. After incubation, the sample was put on ice for 2 min, and then MgCl_2_ (5 mM final) was added to stop the reaction. Excess unbound nucleotide was removed using a NAP-5 column (GE Healthcare, 17085302).

### SOS-catalyzed nucleotide exchange

For studies with mant-nucleotide loaded protein, 12 μL of the protein (1.25 μmol/L) in reaction buffer containing 1 mmol/L of a given incoming nucleotide (GDP or GMPPNP) was added to a 96-well half-area microplate (Corning 3686). After incubation with compounds for 10 min, reactions were initiated by the addition of 3 μL of SOS (10 μmol/L); fluorescence (λ_ex_ = 355 nm, λ_em_ = 460 nm) was monitored for 45 min at 60 s intervals with a multimode microplate reader (PerkinElmer, EnVision). For the exchange assays with incoming mant-nucleotide, 12 μL of the purified protein (1.25 μmol/L) was used (in reaction buffer containing 1 μmol/L of the indicated incoming nucleotide). Fluorescence data were fitted to a one phase decay model with GraphPad Prism 7.0.

### Molecular docking

Compounds were constructed using the Schrödinger Maestro 3D sketcher, and candidate conformations were prepared and generated using LigPrep. Protein structures were optimized using Protein Preparation Wizard software, with docking grids profiles generated around the ligand. Docking was performed using Glide software, with standard precision. The force field was OPLS_2005.

### ITC assays

ITC experiments were carried out at 25 °C with 19 injections, 2 μL per injection, and 150 s intervals using a MicroCal PEAQ-ITC instrument (GE Healthcare). Buffer was exchanged into 25 mM Tris-HCl, 100 mM NaCl, 5 mM MgCl_2_, 1 mM TCEP (Sigma, C4706), 0.05% Tween-20 (Sigma, P1379) and 2.5% (v/v) DMSO. The protein was loaded into a cell, and the compound was titrated. Reference power was set to 10 μcal/s, and the compound solution was titrated at 150 s. Data were fitted into a one site model, and KD, N, Δ*G*, Δ*H*, and −*T*Δ*S* values were calculated using MicroCal Analysis software.

### KRAS–CRAF interaction assay

Three HEK 293T cell lines expressing CRAF together with KRAS(WT), KRAS(G12C), or KRAS(G12D) were generated. Cells were cultured on six-well plates (2.5 × 10^5^ per well) and treated with 1 μg/mL doxycycline for 24 h. Then each well was treated with 200 μL lysis buffer (Promega, E2661) and centrifuged at 4 °C for 5 min. The supernatant was collected and incubated with compounds (TH-Z827, MRTX, or BI-2852) for 10 min. Then 20 μL luciferin substrate buffer (Promega, E2510) was added and the samples were incubated for 10 min before luminescence was measured with a multimode microplate reader (PerkinElmer, EnVision). Data were fitted into an inhibitor-response model to get IC_50_ values.

### 2D cell viability assays

All the cell lines used in this study were obtained from ATCC. PANC-1 and Panc 04.03 cells were seeded onto 96-well microplates (5 × 10^3^ cells per well) and cultured for 24 h with DMEM medium (Gibco, 11960-051) supplemented with 10% FBS (Biological Industries, 04-001-1) and 1% Pen/Strep (Beyotime, C0222). After treatment with compounds (TH-Z827, MRTX, or BI-2852), cells were incubated for another 24 h. Cell Counting Kit-8 reagents (Beyotime, C0042) were added and the samples were incubated for another 1 h. Cell viability was measured at OD (450 nm) using a multimode microplate reader (PerkinElmer, EnVision). Data were fitted into an inhibitor-response model to get IC_50_ values.

### 3D cell viability assays

For comparison of anti-growth activity, a CellTiter-Glo (CTG) 3D cell viability assay (Promega, G9682) was used. Cells (1 × 10^4^ cells per well) were seeded (using the same media) in ultra-low attachment surface 96-well format plates (Corning Costar #3474). On the day after plating, cells were treated with a 7 point three-fold dilution series of indicated compounds (200 μL final volume per well), and cell viability was monitored at 1, 3, 5 days according to the manufacturer’s recommended instructions, after which 50 μL of CellTiter-Glo reagent was added. Samples were then vigorously mixed, covered, and placed on a plate shaker for 20 min to ensure complete cell lysis prior to assessment of luminescent signal.

### Colony formation assay

KPC or PANC1 (1 × 10^3^ cells per well) were seeded in the six-well plates in triplicate. After overnight incubation, cells were treated with various concentrations of TH-Z835 or vehicle (DMSO), and allowed to grow for 10 to 14 days, during which the medium was changed every 3 days. After 10−14 days, cells were fixed by 4% paraformaldehyde (Leagene, DF0135), and cell colonies were stained by crystal violet solution (Beyotime, C0121) for 30 min. After washing with water, 10% methanol-acetic acid solution was added to dissolve the stained cell precipitation, and the absorbance was measured at 590 nm.

### Cell cycle detection by flow cytometry

Cells were seeded in six-well plate and synchronized with serum-free medium for 24 h. Next, the cells were released in a complete medium containing either DMSO or TH-Z835, and collected for analysis at 24 h. For cell cycle analysis, the cell DNA was stained with propidium iodide (PI) using cell cycle and apoptosis analysis kit (Beyotime, C1052). Briefly, cells were harvested by trypsinization and fixed with cold 75% ethanol at 4 °C overnight. The fixed cells were collected and suspended in PBS containing 10 μg/mL PI and 10 μg/mL RNase A, and then incubated at room temperature for 30 min. DNA content was analyzed by the BD FACS Calibur (BD Biosciences), and each histogram was constructed with the data from 10,000 to 20,000 events. The data were analyzed and presented as percentages of total gated cells using the Modfit LT™ Software (BD Biosciences).

### Western blotting

PANC-1 and Panc 04.03 cells were cultured on six-well plates (5 × 10^5^ cells per well) and treated with TH-Z827 for 3 h. Protein samples were prepared, electrophoresed (10% SDS-PAGE), and transferred to a polyvinylidene fluoride membrane. Individual proteins were detected using anti-RAS G12D (Cell Signaling Technology, 14429), anti-pERK1/2 (Cell Signaling Technology, 4370), anti-ERK1/2 (Cell Signaling Technology, 4695), anti-pAKT(Thr308) (Cell Signaling Technology, 2965), anti-pAKT(Ser473) (Cell Signaling Technology, 4060), anti-AKT (Cell Signaling Technology, 2920), anti-tubulin (Proteintech, 66240-1-Ig), anti-EGF Receptor (Cell Signaling Technology, 4267), anti-phospho-EGF Receptor (Tyr1068) (Cell Signaling Technology, 3777), anti-cleaved PARP (Asp214) (Cell Signaling Technology, 9541), anti-cleaved Caspase-3 (Asp175) (Cell Signaling Technology, 9661), anti-cleaved Caspase-7 (Asp198) (Cell Signaling Technology, 9491) antibodies and cell cycle regulation antibody sampler kit (Cell Signaling Technology, 9932). For detection of MAPK signaling in KRAS(G12D) or non-G12D mutant cells, cells were cultured on six-well plates (5 × 10^5^ cells per well) and treated with a serial titration of TH-Z835 or TH-Z827 for 3 h. For apoptosis, RTK feedback regulation, or cell-cycle arrest assay, PANC-1 or KPC were cultured on six-well plates (5 × 10^5^ cells per well) and treated with a serial titration of TH-Z835 at 0, 1, 3, 6, 12, 24, 48, and 72 h (for apoptosis) or at 3, 12, and 24 h (for RTK feedback regulation and cell-cycle arrest assay).

### Apoptosis detection by Annexin V binding

Cells (5 × 10^5^ cells per well) were seeded in six-well plate overnight. Next, the cells were treated with either DMSO or TH-Z835 for 12 h or 24 h. For apoptosis analysis, cells were harvested by trypsinization and washed twice with ice-cold PBS, then stained with Annexin V-FITC and PI by Apoptosis Detection Kit (Beyotime, C1062) in the dark at room temperature for 10 min. Then cells were analyzed with the BD FACS AriaII and FlowJo software.

### Apoptosis detected by WB

For apoptosis, RTK feedback regulation, or cell-cycle arrest assay, PANC-1 or KPC were cultured on six-well plates (5 × 10^5^ cells per well) and treated with a serial titration of TH-Z835 at various time points for up to 72 h.

### Immunogenic cell death and PD-L1 detection by flow cytometry

The cells were treated with TH-Z835 as indicated for 24 h before harvesting. After washing twice in cold PBS, cells were incubated for 30 min with anti-PD-L1 (1:1000, ab213480, Abcam), anti-CRT (1:1000, ab2907, Abcam), or anti-ERp57 (1:1000, ab10287, Abcam) antibody, diluted in cold blocking buffer (2% FBS in PBS), followed by washing and incubation with the Alexa Fluor 488-labeled secondary antibody (1:1000, ZF-0511, ZSGB-BIO) for 30 min. Then cells were analyzed with the BD FACS AriaII and FlowJo software.

### Real-Time quantitative PCR

PANC-1 was treated with multiple doses of TH-Z835 for 24 h. Total RNA was extracted with TRIzol reagent (CWBIO, CW0580). cDNA was prepared using 1 μg of RNA with a cDNA Synthesis Kit (Yeasen, 11141ES10). SYBR-green-based qPCR was performed using primers for PD-L1 (forward, TTTGCTGAACGCCCCATACA; reverse, TTGGTGGTGGTGGTCTTACC), GAPDH (forward, GAGTCAACGGATTTGGTCGT; reverse, TTGATTTTGGAGGGATCTCG). Gene expression was calculated by the comparative ΔΔCT method with the GAPDH for normalization.

### Mouse models

BALB/c nude mice were subcutaneously injected with Panc 04.03 cells (1 × 10^7^ per dose) at Day 0. Mice were randomized when the mean tumor volume was ~70 mm^3^. Each group of mice (*n* = 10) was intraperitoneally injected with PBS, 10 mg/kg or 30 mg/kg TH-Z827 according to the dosage schedule from Day 38 to Day 62.

C57BL/6 mice were subcutaneously injected with KPC (KrasLSL.G12D/ + ; p53R172H/+; PdxCretg/+) cells (5 × 10^5^ per dose). Mice were randomized when the mean tumor volume was ~20 mm^3^. Mice of each group (*n* = 10) were intraperitoneally injected with PBS, anti-PD-1 antibody (100 μg per dose, Bio X Cell, BE0033-2), 10 mg/kg TH-Z827, or a combination (10 mg/kg TH-Z827 and anti-PD-1 antibody) according to a pre-defined dosage schedule from Day 7 to Day 38. Polyclonal Armenian hamster IgG (Bio X Cell, BE0091) was used as a control antibody.

Statistical analysis of differences in mean tumor volume between vehicle and treated groups were assessed using one-way ANOVA test conducted in GraphPad Prism. *P* value < 0.05 was considered statistically significant.

### Immunohistochemistry and immunofluorescence

Tumor samples were obtained after treatment for 30 days, and fixed in 4.0% paraformaldehyde solution (Leagene, DF0135), embedded in paraffin, and cut into 4 μm sections. Sections were used for Immunohistochemistry (IHC) and Immunohistochemistry (IF) according to the standard procedures. The IHC staining protocol was briefly described as follows: the slides were routinely deparaffinized, rehydrated, subjected to antigen retrieval, and incubated in 3% hydrogen peroxide to block endogenous peroxidase. Subsequently, the slides were blocked with 3% BSA and incubated with primary antibodies against pERK1/2 (1:500, Cell Signaling Technology, 4370) or cleaved Caspase-3 (1:400, Cell Signaling Technology, 9661) at 4 °C overnight, and with polymer-HRP-conjugated anti-rabbit secondary antibody (1:200S, Servicebio, GB23303). Then, the sections were stained with DAB kit (Servicebio, G1211), counterstained with hematoxylin, dehydrated, and cover-slipped. For IF staining, slides were incubated with primary antibodies against pERK1/2 (1:200, Cell Signaling Technology, 4370), cleaved Caspase-3 (1:200, Cell Signaling Technology, 9661) at 4 °C overnight, and with CY3-conjugated anti-rabbit secondary antibody (1:300, Servicebio, GB21303). Then, the sections were counterstained with antifading agent DAPI (Beyotime, P0131).

## Supplementary information

Supplementary Material

## Data Availability

Most of the data generated or analyzed during this study are included in this article or available as supplementary information. X-ray crystallographic coordinates and structure factor files have been deposited in the Protein Data Bank (PDB ID: 7EW9, 7EWA, 7EWB). Other data that support the findings of this study are available from the corresponding authors upon request.
