# Supplementary material for: KRAS(G12D) can be targeted by potent inhibitors via formation of salt bridge
Source: Cell Discov. 2022 Jan 25;8:5. doi: 10.1038/s41421-021-00368-w (PMC8786924; doi:10.1038/s41421-021-00368-w)
Supplement: Supplementary file 1 — Supplementary Material [file 41421_2021_368_MOESM1_ESM.pdf]

KRAS(G12D) can be targeted by potent inhibitors via formation of salt bridge

Zhongwei Mao[1], Hongying Xiao[1,2], Panpan Shen[3], Yu Yang[3], Jing Xue[1], Yunyun Yang[1], Yanguo Shang[1], Lilan Zhang[3], Xin Li[1,2], Yuying Zhang[4], Yanan Du[4], Chun-Chi Chen[3], Rey-Ting Guo[3], Yonghui Zhang[1]

[1]School of Pharmaceutical Science, Tsinghua-Peking Center for Life Sciences, Tsinghua University; Beijing Advanced Innovation Center for Human Brain Protection, Beijing, China.

[2]Joint Graduate Program of Peking-Tsinghua-NIBS, School of Life Sciences, Tsinghua University, Beijing, China.

[3]State Key Laboratory of Bio-catalysis and Enzyme Engineering, Hubei Collaborative Innovation Center for Green Transformation of Bio-Resources, School of Life Sciences, Hubei University, Wuhan, China.

[4]Department of Biomedical Engineering, School of Medicine, Tsinghua-Peking Center for Life Science, Tsinghua University, Beijing, China.

These authors contributed equally: Zhongwei Mao, Hongying Xiao, Panpan Shen, Yu Yang.

Correspondence: Rey-Ting Guo (guoreyting@hubu.edu.cn) or Yonghui Zhang (zhangyonghui@tsinghua.edu.cn)

Table S1 Crystallization data collection and refinement statistics

	KRAS G12D TH-Z816	KRAS G12D TH-Z827	KRAS G12D TH-Z835
PDB code	7EW9	7EWA	7EWB
Data collection			
Space group	$P12_11$	$P12_11$	$P12_11$
Cell dimensions			
a, b, c (Å)	47.0, 103.4, 56.3	46.7, 102.8, 56.4	46.6, 102.5, 56.1
α, β, γ (º)	90, 106.4, 90	90, 107.1, 90	90, 106.9, 90
Resolution (Å)	50.0 – 2.13 (2.16 – 2.13)	50.0 – 2.25 (2.28 – 2.25)	50.0 – 1.99 (2.02 – 1.99)
No. of observed reflections	28858 (1177)	24193 (946)	34543 (1505)
Redundancy	7.0 (4.0)	9.1 (6.7)	6.1 (3.5)
Completeness (%)	99.9 (98.5)	100.0 (100.0)	99.9 (100.0)
I/sigma (I)	14.1 (2.4)	12.6 (3.3)	10.0 (2.1)
R_{merge} (%)[b]	7.2 (42.0)	8.4 (39.8)	8.8 (39.5)
$CC_{1/2}$	1.0 (0.81)	1.0 (0.95)	1.0 (0.88)
Refinement[c]			
R_{work}	20.1	20.3	19.5
R_{free}	25.7	25.8	24.6
r.m.s.d bonds (Å)	0.008	0.008	0.007
r.m.s.d angles (º)	1.098	1.079	1.105
Ramachandran statistics			
Preferred (%)	96.8	97.3	98.0
Allowed (%)	3.0	2.7	2.0
Outliers (%)	0.2	0	0
Average B-factor (Å²) / Atoms			
Protein	39.1 / 4015	43.6 / 3902	31.1 / 4053
Mg	36.5 / 3	42.0 / 3	30.8 / 3
GMPPNP or GDP	31.8 / 84	37.9 / 92	26.7 / 92
Inhibitor	40.1 / 72	43.4 / 74	32.4 / 111
Solvent	41.3 / 276	43.5 / 169	34.4 / 317

[a] Values in parentheses are for the highest resolution shell.

[b] $R_{merge} = \Sigma_{hkl}\Sigma i\ |I_i(hkl) - \langle I(hkl)\rangle| / \Sigma_{hkl}\Sigma_i I_i(hkl)$, where the sum is the overall *i* measured reflections with equivalent miller indices *hkl*; $\langle I(hkl)\rangle$ is the averaged intensity of these *i* reflections, and the grand sum is the overall measured reflections in the data set.

[c] All positive reflections were used in the refinement.

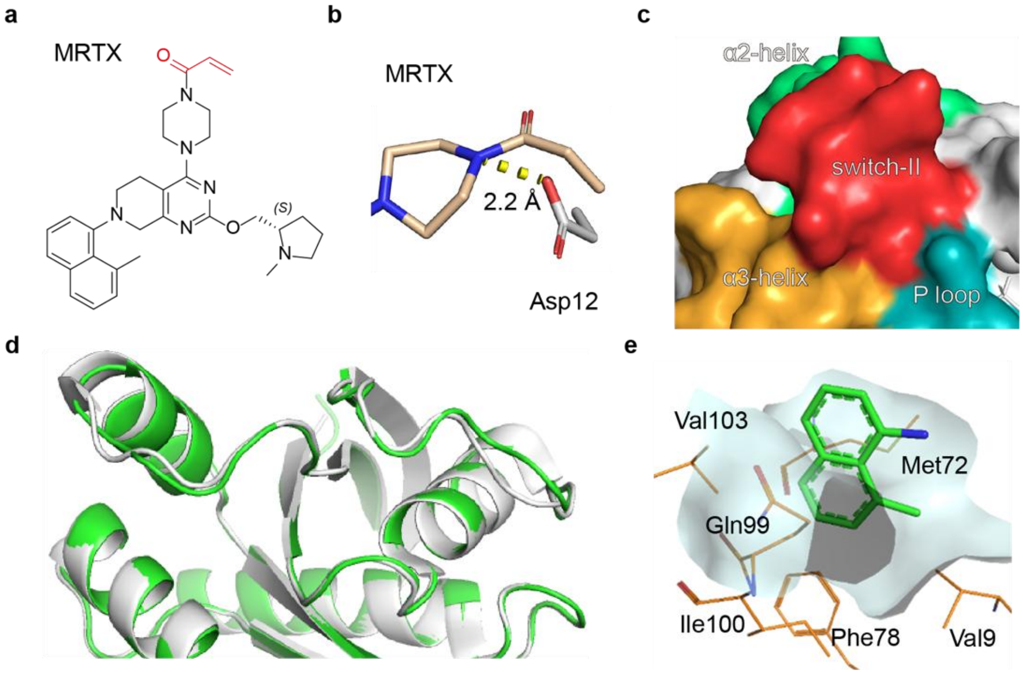

Fig. S1: Additional insights into compound binding and the induced-fit pocket of KRAS(G12D). a Chemical structure of the previously reported acryloyl-moiety-containing KRAS(G12C) inhibitor MRTX (the acryloyl moiety is highlighted in red). **b** Docking pose showing the 2.2 Å distance between the N atom of piperazine moiety (PDB ID: 6USX) and the O atom of Asp12 (PDB ID 4EPR). **c** The inhibitor-free KRAS(G12D) structure (PDB ID: 4EPR). Secondary structures, including α2-helix (green), switch II (red), α3-helix (orange) and P loop (teal), are shown as a surface diagram. **d** Alignment of TH-Z816-bound KRAS(G12D) (green, PDB ID: 7EW9) and MRTX-bound KRAS(G12C) (white, PDB ID: 6USX), shown as secondary structures. **e** There is a hydrophobic pocket around the naphthyl moiety of TH-Z816, comprising Val9, Met72, Phe78, Gln99, Ile100, and Val103.

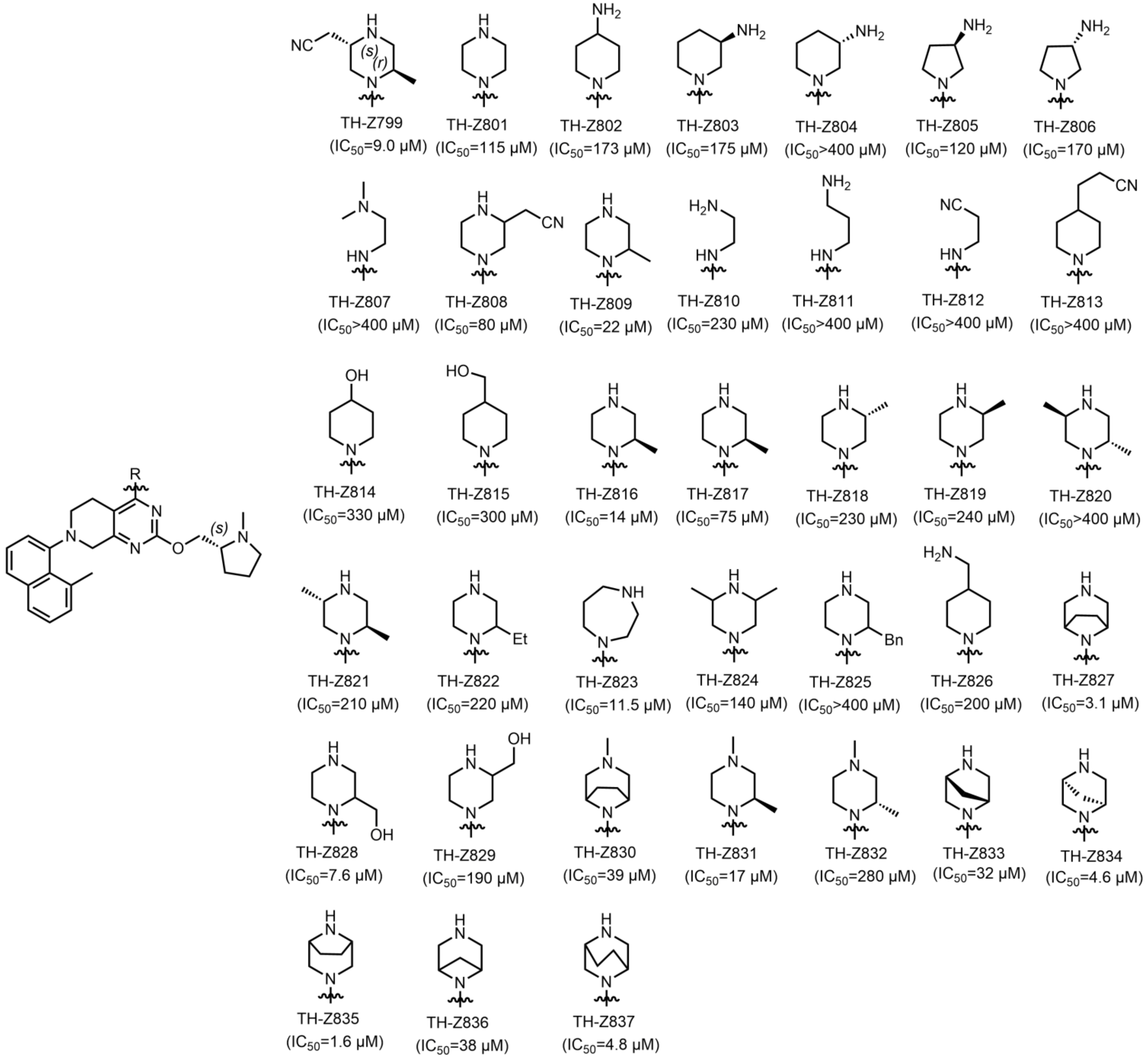

Fig. S2: Chemical structures of KRAS(G12D) inhibitors with various R substituents and their inhibitory activities tested by SOS-catalyzed nucleotide exchange assay.

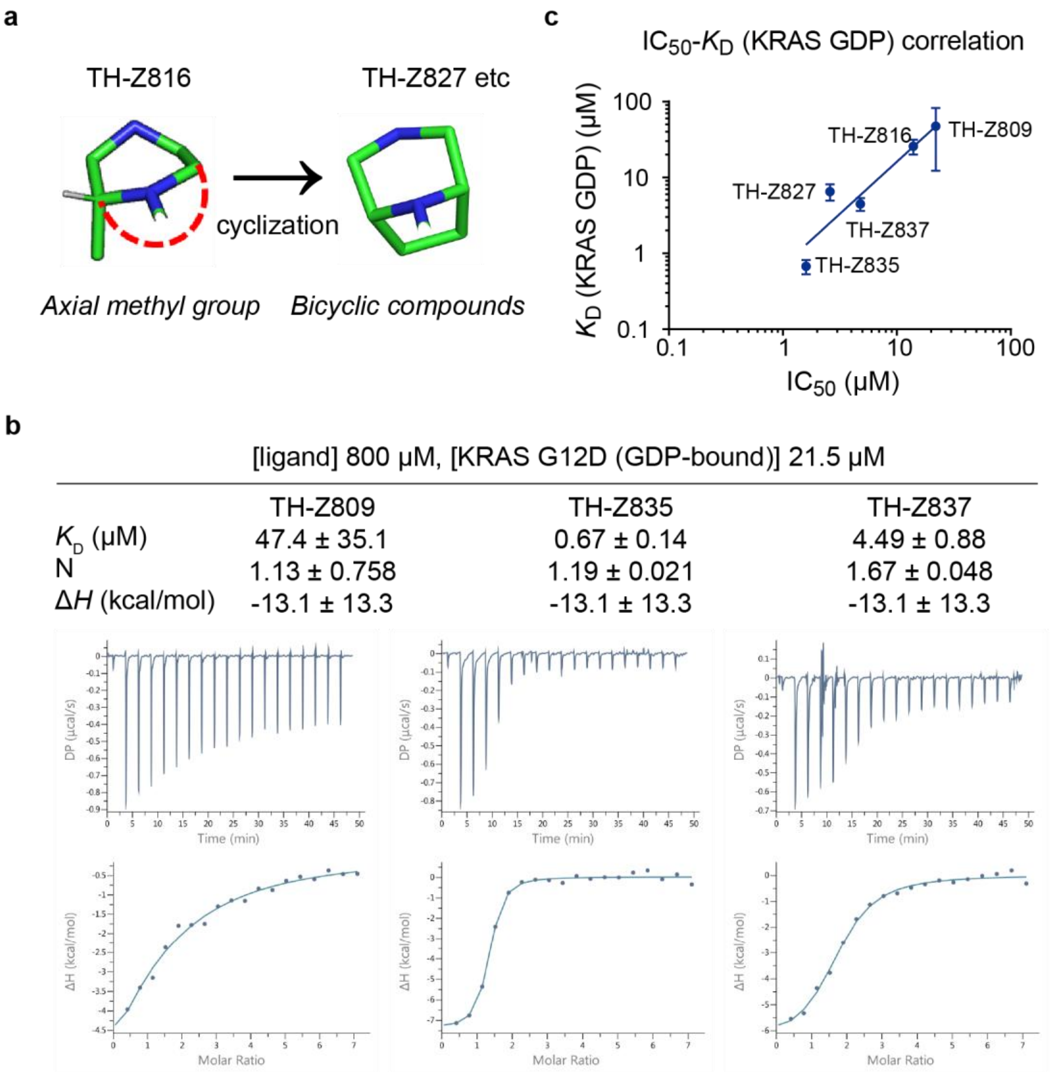

Fig. S3: Structural analysis of KRAS(G12D) bound to TH-Z816. a The axial position of the methyl group of TH-Z816 suggests cyclization as a feasible strategy for inhibitor design. **b** ITC assays of the indicated compounds (800 μM) and GDP-bound KRAS(G12D) (21.5 μM). **c** ITC K_D (GDP-bound KRAS(G12D)) and IC_{50} values of the indicated compounds, fit based on linear correlation (blue line). The structures of all compounds are shown in Supplementary **Fig. S2**.

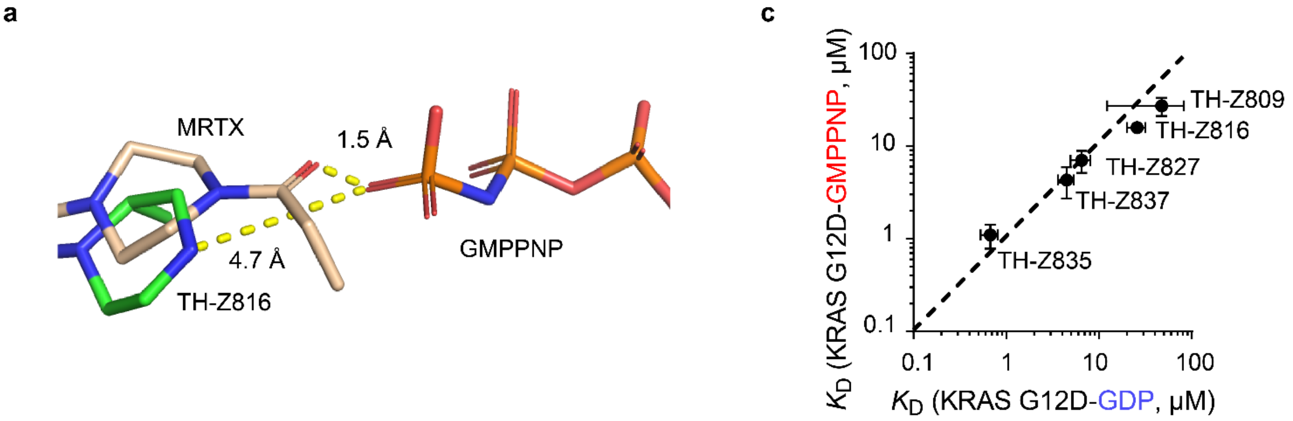

b

	TH-Z809	TH-Z816	TH-Z827	TH-Z835	TH-Z837
[ligand] 800 μM					
[KRAS G12D (GMPPNP-bound)]	29 μM	29 μM	47 μM	47 μM	47 μM
K_D (μM)	27.1 ± 6.03	15.8 ± 1.36	7.01 ± 1.88	1.10 ± 0.319	4.31 ± 1.60
N	1.00	0.73 ± 0.032	1.09 ± 0.04	1.01 ± 0.022	1.22 ± 0.052
Δ*H* (kcal/mol)	-7.82 ± 0.995	-11.0 ± 0.749	-5.96 ± 0.438	-6.06 ± 0.227	-6.06 ± 0.486
-*T*ΔS (kcal/mol)	1.59	4.46	-1.06	-2.07	-1.27
Δ*G* (kcal/mol)	-6.23	-6.55	-7.03	-8.13	-7.32

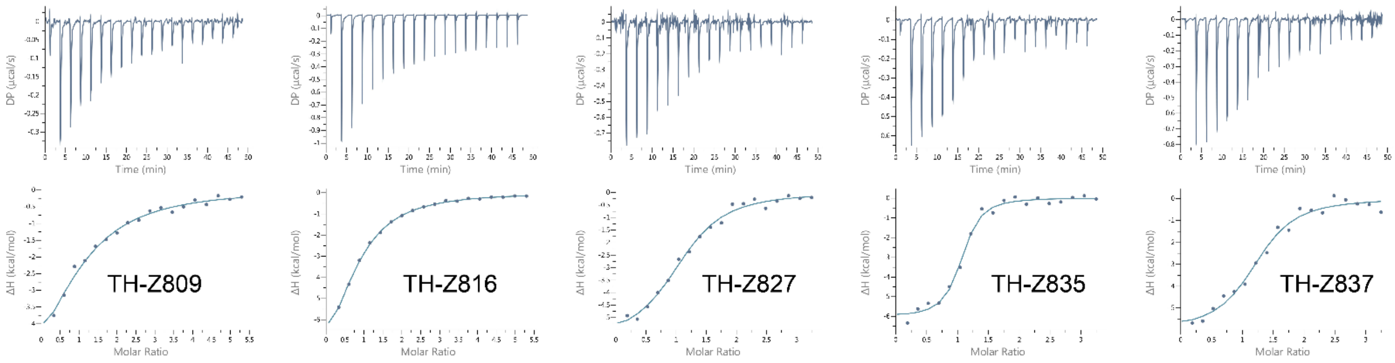

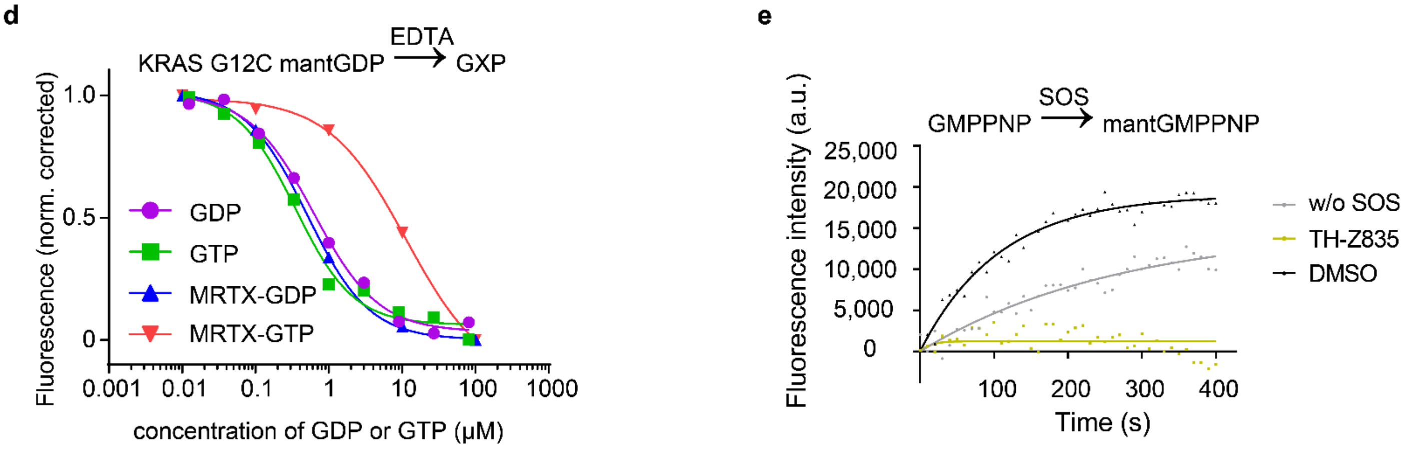

Fig. S4: Further analysis of the binding mode of GMPPNP-bound KRAS. a Computational modeling indicates our G12D inhibitor (PDB ID: 7EW9) does not have steric clash with the γ-phosphate of GMPPNP (PDB ID: 5USJ). The distance between the γ-phosphate of GMPPNP (PDB ID: 5USJ) and the acryloyl moiety of the G12C inhibitor MRTX (PDB ID: 6USX) is 1.5 Å. The distance between the γ-phosphate of GMPPNP and the piperazine moiety of the G12D inhibitor TH-Z816 (PDB ID: 7EW9) is 4.7 Å. The protein structure was modeled using the Protein Preparation Wizard of Schrödinger Maestro. **b** ITC assay of each compound with GMPPNP-bound KRAS(G12D). **c** ITC K_D values for each compound for both GMPPNP-bound and GDP-bound KRAS(G12D). The dashed line is a linear fitting line (y = x). **d** EDTA-mediated competition between fluorescently labeled mantGDP loaded on KRAS and free nucleotide (GDP or GTP). The experiment was carried out with KRAS(G12C) alone (1 μM) or with KRAS(G12C) treated with MRTX (3 μM). **e** Inhibitory activity of TH-Z835 measured by SOS-catalyzed nucleotide exchange assays with mantGMPPNP as the incoming nucleotide.

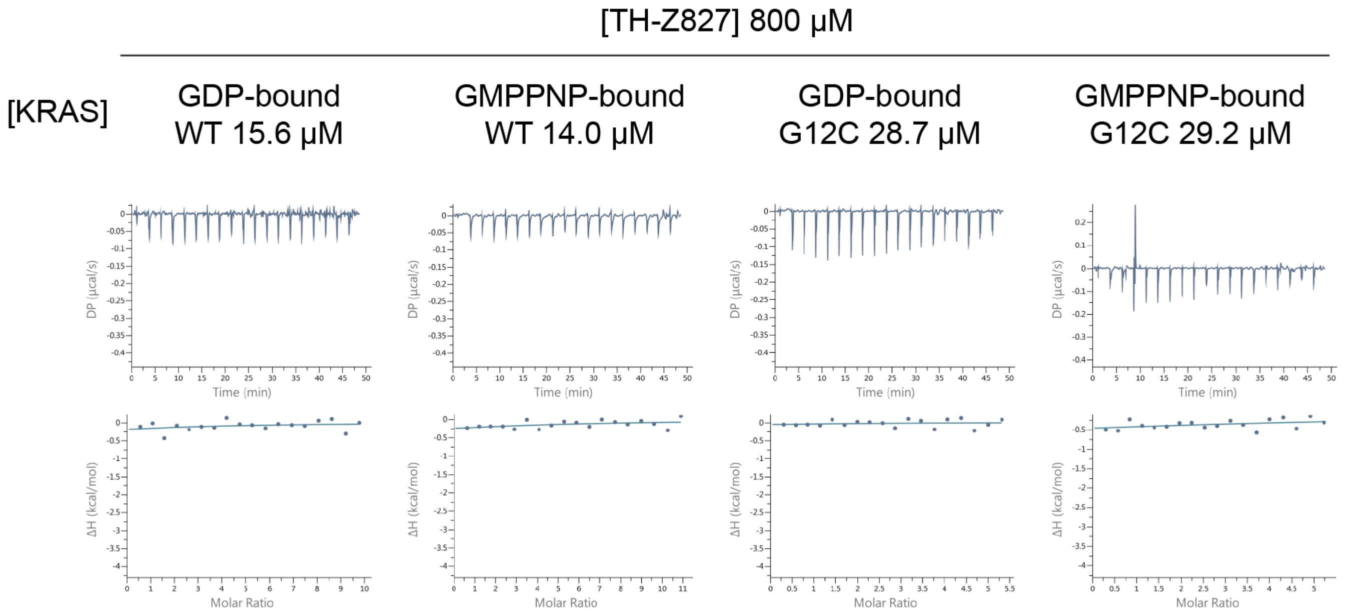

Fig. S5: Binding assay of TH-Z827 with GDP- or GMPPNP-bound KRAS (WT or G12C). ITC assays of TH-Z827 (800 µM) and GDP- or GMPPNP-bound KRAS (WT or G12C).

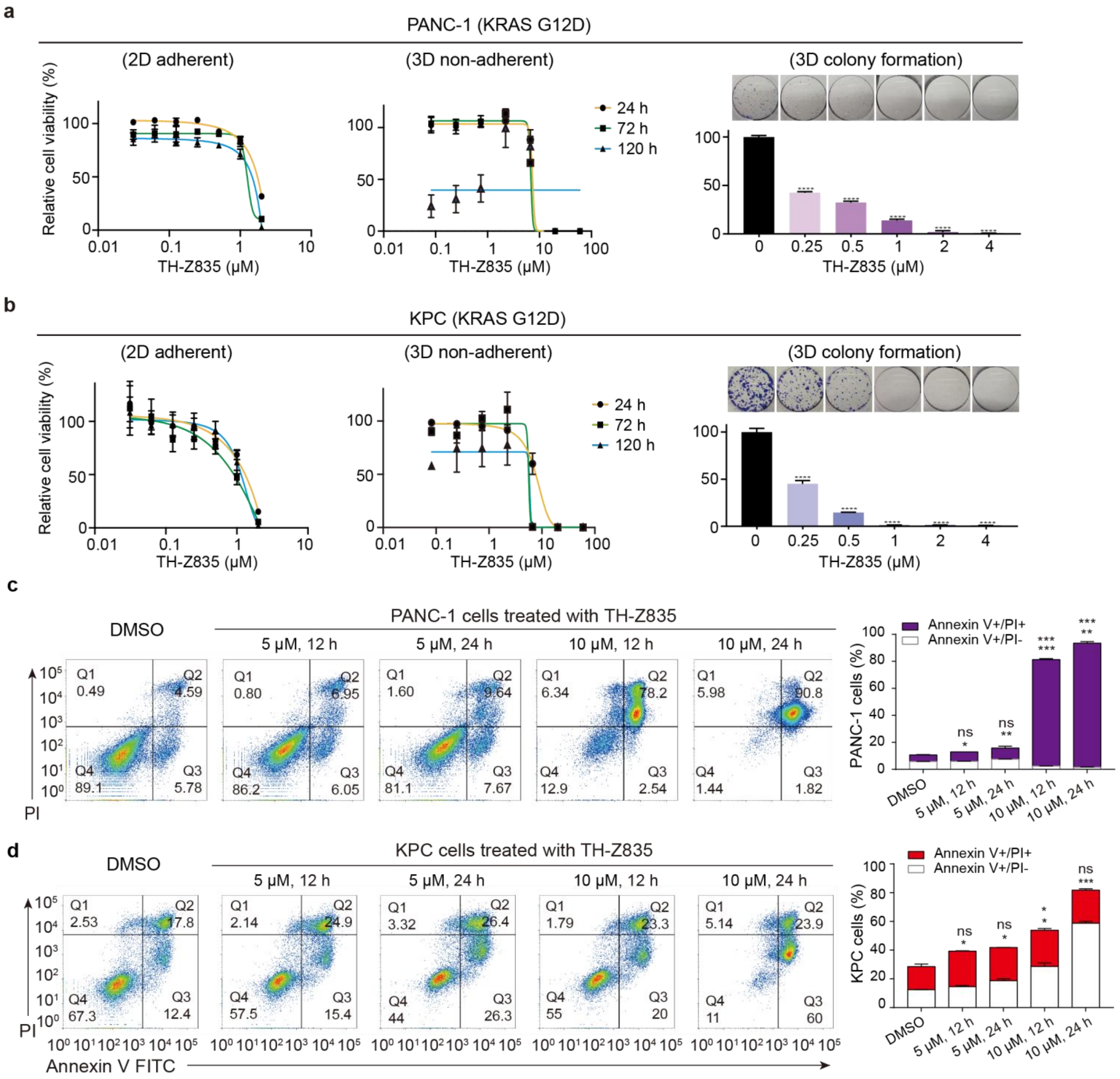

Fig. S6: Anti-proliferative effects and signaling inhibition of KRAS(G12D) inhibitors. a, b Cell viability assays of PANC-1 (**a**) and KPC (**b**) cells treated with indicated concentration of TH-Z835 for 24 h, 72h, or 120 h in 2D adherent assays (left panel) and 3D non-adherent assays plates (middle panel). As for colony formation assay (right panel), PANC-1 cells were cultured for 14 days and KPC cells were cultured for 10 days. Data are shown as means ± SEM (n = 3), two-tailed Student's t-test, **** P < 0.001. **c, d** Left panel: Cell apoptosis analyzed by flow cytometry of

PANC-1 (**c**) or KPC (**d**) cells upon a 12-h or 24-h treatment with TH-Z835 (5 μM or 10 μM). Right panel: apoptotic (Annexin V-positive) cell proportions were quantified. Data are show as means ± SEM (*n* = 3), two-tailed Student's t-test, * *P* < 0.05, ** *P* < 0.01, *** *P* < 0.001.

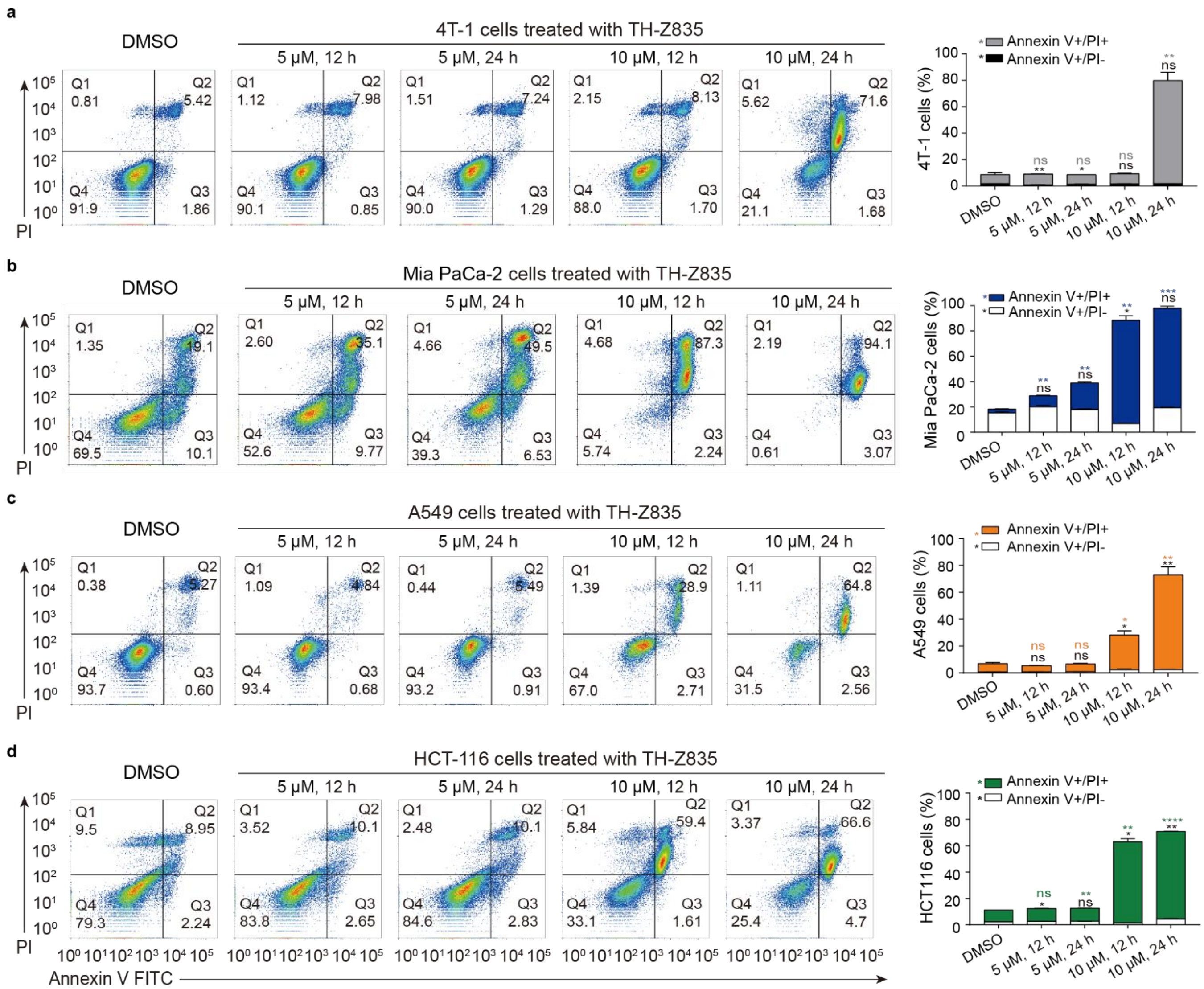

Fig. S7: TH-Z835 induces apoptosis in different KRAS mutant cells.

Left panel: apoptosis analysis by flow cytometry of 4T1 (**a**), MIA PaCa-2 (**b**), A549 (**c**), and HCT116 (**d**) cells upon a 12-h or 24-h treatment of TH-Z835. Right panel: Apoptotic (Annexin V-positive) cell proportions were quantified. Data are shown as the means ± SEM (*n* = 3), two-tailed Student's *t*-test, * *P* < 0.05, ** *P* < 0.01, *** *P* < 0.001, **** *P* < 0.0001.

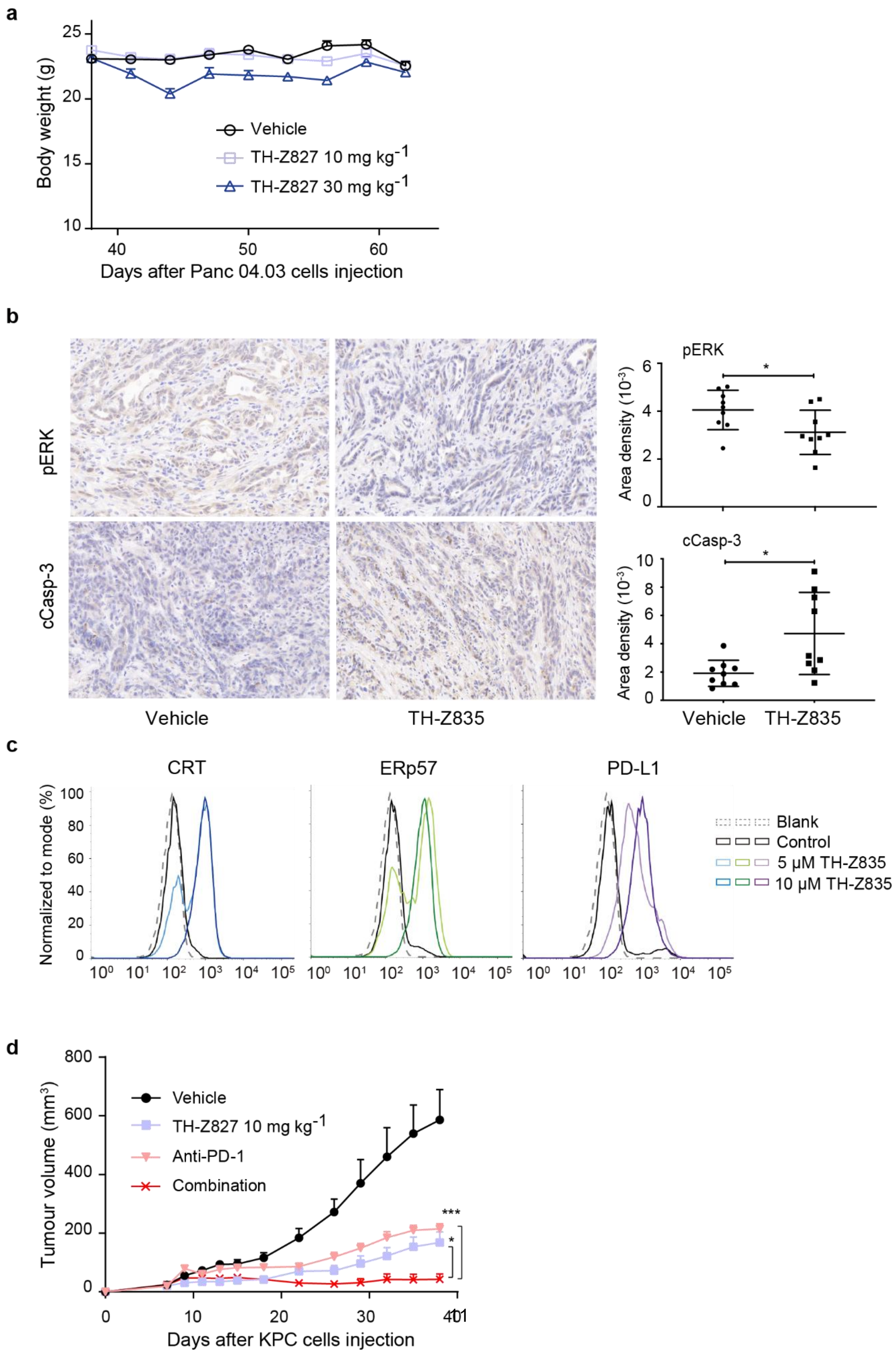
a
Body weight (g)
Vehicle
TH-Z827 10 mg kg-1
TH-Z827 30 mg kg-1
Days after Panc 04.03 cells injection
b
pERK
cCasp-3
Vehicle
TH-Z835
Area density (10-3)
c
CRT
ERp57
PD-L1
Normalized to mode (%)
Blank
Control
5 μM TH-Z835
10 μM TH-Z835
d
Tumour volume (mm3)
Vehicle
TH-Z827 10 mg kg-1
Anti-PD-1
Combination
Days after KPC cells injection

Fig. S8: Anti-tumor effects of the KRAS (G12D) inhibitors alone and in combination with anti-PD-1 antibody

a Body weight (means ± SEM, n = 10) of mice bearing xenograft tumors (from inoculation of Panc 04.03 cells) treated intraperitoneally with TH-Z827 at 10 mg kg^{-1} or 30 mg kg^{-1}. **b** Left panel: Immunohistochemical (IHC) analysis of pERK and cleaved Caspase-3 in tumor section. Scale bar, 20 μm. Right panel: Quantifications of IHC positive staining (means ± SEM, n = 9) were analyzed using two-tailed Student's *t*-test, * $P < 0.05$. **c** Flow cytometry analysis of PD-L1 and immunogenic cell death (ICD) markers (CRT and ERp57) on the surface of KPC cells after 24-h treatment with TH-Z835. **d** C57BL/6 mice were injected with KPC cells at Day 0, after which TH-Z827, anti-PD-1 antibody, or a combination therapy (10 mg kg-1 TH-Z827 and 100 μg per dose anti-PD-1 antibody) was IP administered using the same dosage schedule shown in **Fig. 7f**. Combination treatment (n = 5, shown as the mean ± SEM) led to a statistically significant decrease in tumour volumes at day 38 compared with either single-agent treatment (one-way ANOVA followed by Dunnett's test; * $P_{adj} < 0.05$, *** $P_{adj} < 0.001$)..
